# Anti-Inflammatory Effects of Alpha-Lipoic Acid Modulate Cystathionine-γ-Lyase Expression in RAW 264.7 Macrophages

**DOI:** 10.3390/ijms27020949

**Published:** 2026-01-18

**Authors:** Aqsa Shahid, Stephen Chambers, Amy Scott-Thomas, Masuma Zawari, Madhav Bhatia

**Affiliations:** Department of Pathology and Molecular Medicine, University of Otago, Christchurch 8140, New Zealand; shaaq042@student.otago.ac.nz (A.S.); masuma.zawari@otago.ac.nz (M.Z.)

**Keywords:** Alpha-lipoic acid, cystathionine-γ-Lyase, LPS-stimulated macrophages, anti-inflammatory agent, antioxidant agent

## Abstract

Alpha-lipoic acid (ALA) is a naturally occurring organosulfur compound with antioxidant and anti-inflammatory activities. The time-dependent effects of ALA and mechanism of interaction with cystathionine-γ-lyase (CSE—an enzyme responsible for hydrogen sulfide—H_2_S synthesis) in RAW 264.7 macrophages remain unknown. In this study, we report results supporting the hypothesis that anti-inflammatory effects of ALA are associated with the reduction in CSE expression. To investigate the temporal effect of ALA in lipopolysaccharide (LPS—a potent stimulator of inflammation) treated RAW 264.7 macrophages, ALA was administered 1 h before LPS stimulation and 1, 3, and 6 h post LPS stimulation. Effects of ALA on different inflammatory and oxidative stress biomarkers including tumor necrosis factor-alpha (TNF-α), interleukin-6 (IL-6), monocyte chemoattractant protein-1 (MCP-1), catalase activity (CAT), and malondialdehyde (MDA) levels were investigated. LPS stimulation significantly increased TNF- α, IL-6, MCP-1, MDA levels, and CSE expression and decreased CAT activity compared with the control group (*p* < 0.05 to 0.0001). ALA treatment at 1000 µM significantly attenuated LPS-stimulated inflammatory response in the macrophages across different time points (*p* < 0.05 to 0.0001). Furthermore, we found that ALA treatment reduced the expression of CSE in both pre- and post-treated LPS-stimulated macrophages in a time-dependent manner. In conclusion, this study demonstrated for the first time that the protective effects of ALA are dependent on the reduction in CSE expression in LPS-stimulated RAW 264.7 macrophages.

## 1. Introduction

Inflammation is a protective response of the body against external stimuli characterized by the production of inflammatory mediators. As an important component of the innate immune system, macrophages play a vital role against microbial invasion [1]. During pathological conditions, the overproduction of inflammatory mediators by macrophages initiates a cascade of inflammatory reactions. Excessive production of these inflammatory mediators can lead to several pathological conditions, including sepsis, acute pancreatitis (AP), inflammatory bowel disease (IBD), rheumatoid arthritis (RA), and atherosclerosis [2]. There is an urgent need to develop innovative therapeutic strategies to control inflammation in various disease conditions.

LPS is a major component of the Gram-negative bacterial cell wall and a potent stimulator of inflammation [3]. RAW 264.7 mouse macrophages are an ideal in vitro model, that are widely used to study LPS-induced inflammation. LPS-stimulated RAW 264.7 macrophages trigger the activation of several pro-inflammatory mediators including the gasotransmitter hydrogen sulfide (H_2_S), the chemokine MCP-1, and cytokines such as TNF-α and IL-6 [4,5]. Toll-like receptor 4 (TLR4), present on the immune cells, contributes to the recognition of LPS and initiates the intracellular signaling cascade, which triggers the inflammatory responses in cells [6]. An imbalance between the generation and accumulation of reactive oxygen species (ROS) affects the ability of cells and tissues to neutralize these harmful products [7]. Furthermore, LPS stimulation also results in the excessive production of ROS, which can be evidenced by changes in CAT activity and MDA levels [8,9].

ALA, an organosulfur antioxidant (also called thioctic acid), was originally isolated from bovine liver in 1951 [10]. ALA is predominantly present in mitochondrial enzymes and serves as a cofactor for the α-ketoglutarate dehydrogenase complex (KDC) and pyruvate dehydrogenase complex (PDC) [11]. Due to its potent antioxidant activities, ALA can reduce the generation of ROS and alleviate disease symptoms [12]. A substantial body of evidence suggests the antioxidant and anti-inflammatory effects of ALA, whereas the molecular mechanisms underlying the protective effects of ALA remain unknown. In the past, H_2_S was merely considered as a toxic gas, but recent research has unveiled it serves as an important signaling molecule with physiological functions and contributing to several pathological conditions. A few studies have suggested the protective effects of ALA could be associated with the formation of sulfane sulfur (source of H_2_S formation). It has been reported that administration of ALA leads to the formation of dihydrolipoic acid (DHLA—reduced form), which triggers H_2_S release through sulfane sulfur metabolism in the rat liver [13].

As the cellular mechanisms by which ALA acts are not clearly known, we have investigated the mechanism by which ALA treatment could protect against inflammation in an in vitro model of LPS-stimulated RAW 264.7 macrophages. These cells were treated with ALA both before and after LPS stimulation to investigate the effect of ALA prophylactic and therapeutic treatment on the inflammatory response.

## 2. Results

### 2.1. Effect of ALA Treatment on LPS-Stimulated Chemokine Production by RAW 264.7 Macrophages as a Function of ALA Concentration

**Figure 1:**
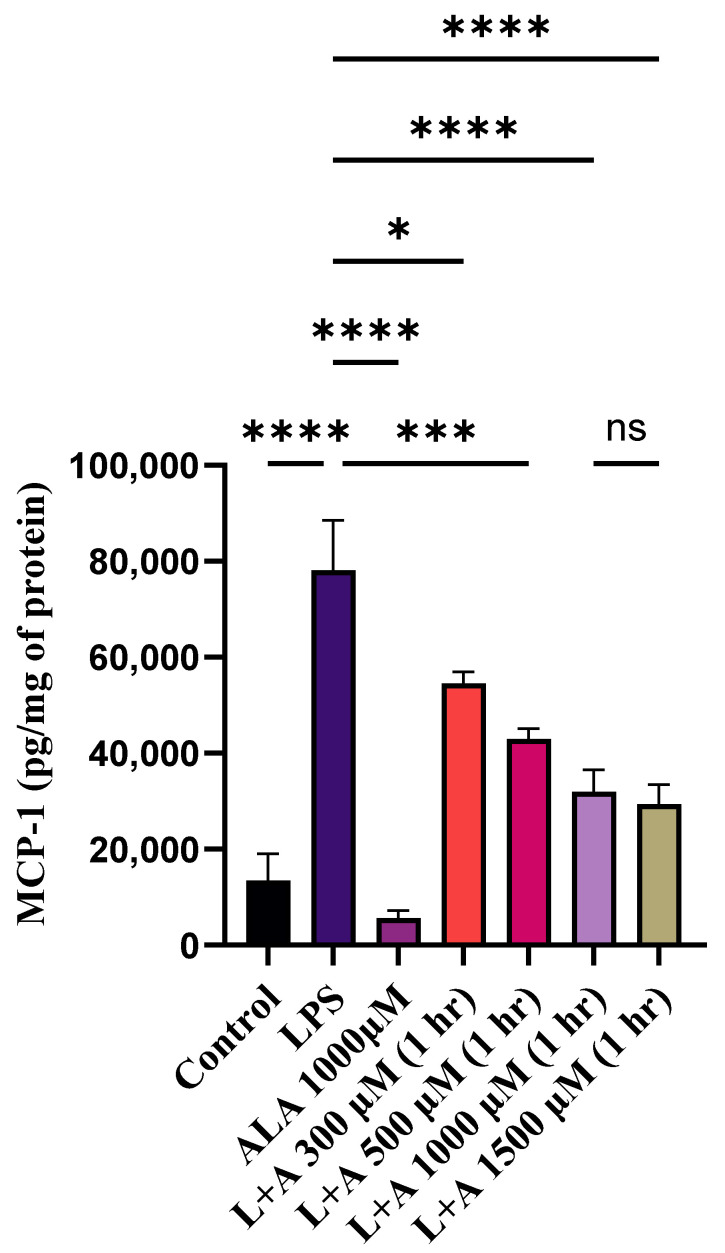
Dose-dependent assay for ALA in LPS-stimulated macrophages. LPS stimulation significantly increased the release of MCP-1. Maximum protective effect was observed at 1000 µM and 1500 µM ALA. For subsequent in vitro experiments, 1000 µM was selected as an optimal concentration. Results are expressed in pg/mg of protein and represented as mean ± SEM (*n* = 3). Each experiment was performed in triplicate on three different days. A one-way ANOVA with a post hoc Tukey test was used. Statistical significance was determined at * *p* < 0.05; *** *p* < 0.001; **** *p* < 0.0001; and non-significant (ns).

To check the anti-inflammatory effects of ALA, LPS-stimulated macrophages were treated with ALA (Figure 1). To determine the optimal concentration of ALA for subsequent experiments, cells were treated with 300 µM, 500 µM, 1000 µM, and 1500 µM ALA. MCP-1 was selected as a representative biomarker for dose optimization, and its concentration was measured by ELISA. The maximum protective effect was observed at 1000 µM, and no further effect was observed with ALA at a higher concentration. Therefore, 1000 µM was selected as an optimal concentration for subsequent in vitro experiments.

### 2.2. Treatment of ALA Inhibits LPS-Induced Up-Regulation of MCP-1, TNF-α, and IL-6 in RAW 264.7 Macrophages

**Figure 2:**
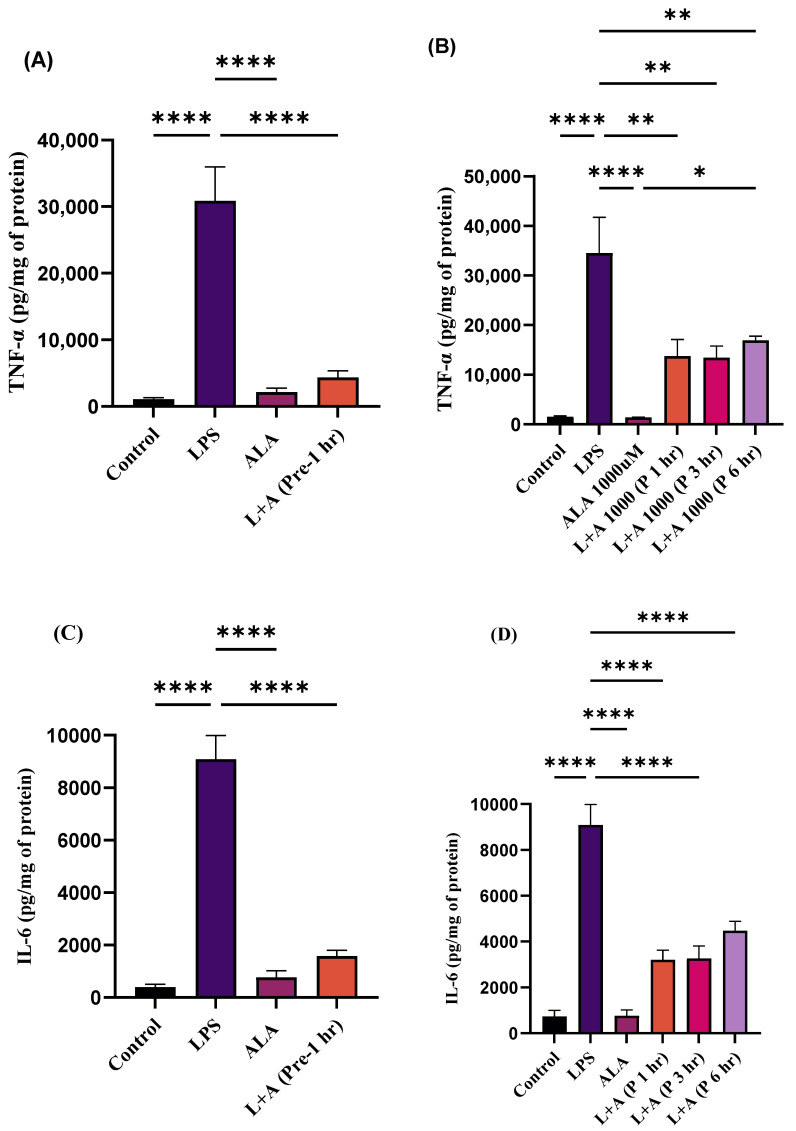

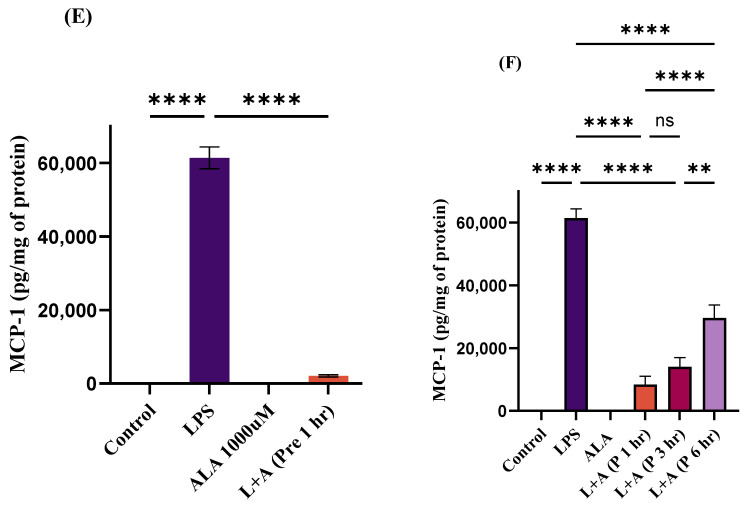
(**A**–**F**) Effect of ALA treatment either 1 h before (pre-treatment) or 1, 3, and 6 h post LPS stimulation in RAW 264.7 macrophages. LPS stimulation significantly increased the levels of TNF-α, IL-6, and MCP-1. Pre- and post-treatment with ALA in LPS-stimulated RAW 264.7 macrophages significantly reduced the release of MCP-1, TNF-α, and IL-6. Results are expressed in pg/mg of protein and represented as mean ± SEM (*n* = 3). Each experiment was performed in triplicate on three different days. A one-way ANOVA with a post hoc Tukey test was used. Statistical significance was determined at * *p* < 0.05; ** *p* < 0.01; **** *p* < 0.0001; and non-significant (ns). The quantitative ELISA data are provided in Supplementary Table S1.

Treatment with LPS significantly increased the production of pro-inflammatory mediators (cytokines and chemokines) in RAW 264.7 macrophages (Figure 2A–F). However, increases in levels of these pro-inflammatory mediators were significantly attenuated by ALA pre-treatment (Figure 2A–F). Furthermore, the increases in the levels of these pro-inflammatory mediators were attenuated in a time-dependent manner by ALA treatment after LPS stimulation (Figure 2A–F). These findings highlight the anti-inflammatory effect of ALA in LPS-stimulated RAW 264.7 macrophages.

### 2.3. Effect of ALA Treatment on the Morphology of LPS-Stimulated Macrophages

**Figure 3:**
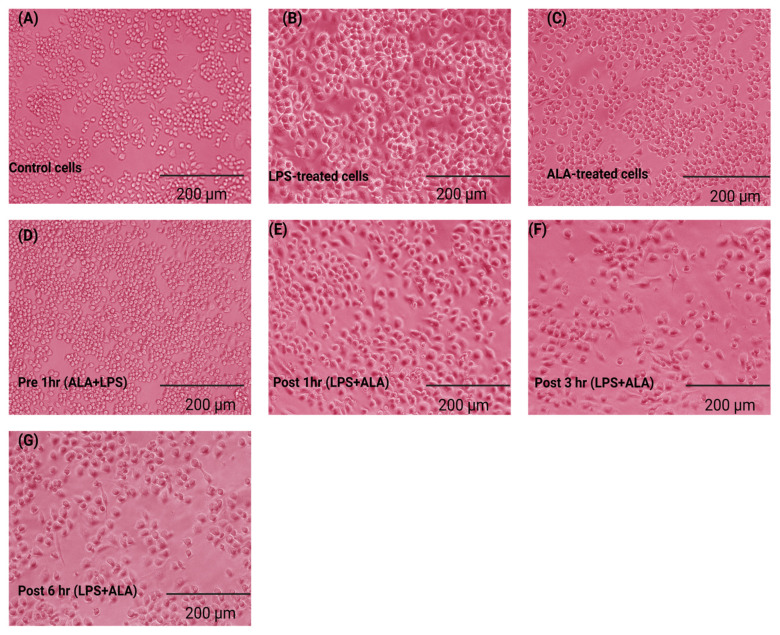
Representative image from one experiment to show protective effects of ALA on the morphology of LPS-stimulated RAW 264.7 macrophages (scale bar = 200 µm). (**A**) Normal RAW 264.7 macrophages. (**B**) LPS-stimulated RAW 264.7 macrophages, 100 ng/mL. (**C**) Treatment of RAW 264.7 macrophages with 1000 μM ALA. (**D**) ALA pre-treatment at 1 h before LPS stimulation in RAW 264.7 macrophages. (**E**) ALA post-treatment at 1 h after LPS stimulation in RAW 264.7 macrophages. (**F**) ALA post-treatment at 3 h after LPS stimulation in RAW 264.7 macrophages. (**G**) ALA post-treatment at 6 h after LPS stimulation in RAW 264.7 macrophages. A simple ImageJ (1.54p, National Institutes of Health, Bethesda, MD, USA)-based morphometric analysis was performed to support the qualitative analysis of cells. Raw data are provided in Supplementary Excel File S1.

The effects of ALA on macrophage activation were assessed by evaluating the morphological changes in LPS-stimulated RAW 264.7 macrophages. Normal macrophages have a round or oval shape with smooth edges and surfaces (Figure 3A). After 24 h of LPS stimulation, several hallmarks of macrophage activation, including irregular polygonal shape, extension of pseudopodia on the cell surface, cellular hypertrophy, increased intracytoplasmic vacuolation, and accumulation of granules, were observed (Figure 3B). These morphological changes in macrophages showed that exposure to LPS resulted in a severe inflammatory response. Cells treated with ALA only did not exhibit any cytotoxic effects or morphological alterations (Figure 3C). Treatment with ALA significantly preserved the morphological alterations in LPS-stimulated macrophages. Pre-treatment with ALA for 1 h before LPS stimulation prevented the morphological changes induced by LPS (Figure 3D). Furthermore, post-treatment with ALA exhibited a time-dependent protective effect against LPS-induced cell injury. Post-treatment with ALA 1 h after LPS stimulation preserved cell morphology with minimal vacuolation and intracytoplasmic granules (Figure 3E). Post-treatment with ALA at 3 and 6 h, macrophages showed high vacuolation, rough edges, increased cytoplasmic granulation, and hypertrophy (Figure 3F,G). These findings indicate that the effect of ALA in response to LPS exposure is time-dependent.

### 2.4. Effect of ALA Treatment on Catalase (CAT) Activity in LPS-Stimulated Macrophages

**Figure 4:**
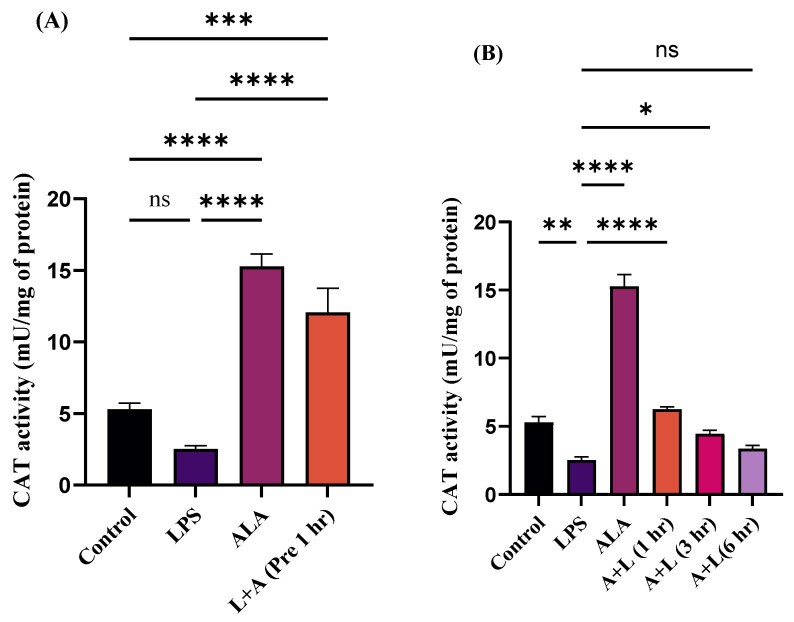
(**A**,**B**) Effect of ALA treatment on CAT activity either 1 h before (pre-treatment) or 1, 3, and 6 h post LPS stimulation in RAW 264.7 macrophages. Pre- and post-treatment with ALA in LPS-stimulated RAW 264.7 macrophages increased the CAT activity. Maximum restoration was observed at 1 h ALA pre-treatment. Effects of ALA on CAT activity are strongly time-dependent. Results are expressed in mU/mg of protein and represented as mean ± SEM (*n* = 3). Each experiment was performed in triplicate on three different days. A one-way ANOVA with a post hoc Tukey test was used. Statistical significance was determined at * *p* < 0.05; ** *p* < 0.01; *** *p* < 0.001; **** *p* < 0.0001; and non-significant (ns). The quantitative CAT activity data are provided in Supplementary Table S1.

To evaluate the antioxidant effects of ALA, CAT activity was measured in LPS-stimulated macrophages (Figure 4A,B). LPS markedly decreased the CAT activity in macrophages, whereas treatment with ALA prevented this effect. Pre-treatment with ALA showed a significant increase in antioxidant protection similar to that of ALA alone in LPS-stimulated macrophages (Figure 4A). Post-treatment with ALA at 1 h partially restored CAT activity. The mean CAT activity was higher compared to the LPS group, but the effect was significantly lower than that observed with ALA pre-treatment. Post-treatment of ALA at 3 and 6 h did not restore the mean CAT levels in LPS-stimulated macrophages to control levels (Figure 4B). These findings highlight that ALA exhibits maximum protective effects before LPS stimulation, and these effects diminished progressively over time.

### 2.5. Effect of ALA Treatment on Lipid Peroxidation in LPS-Stimulated Macrophages

**Figure 5:**
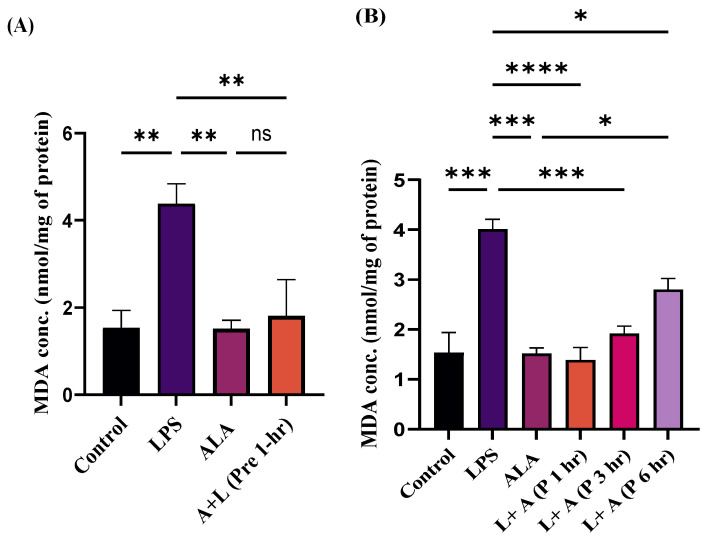
(**A**,**B**) Effect of ALA treatment on lipid peroxidation either 1 h before (pre-treatment) or 1, 3, and 6 h post-stimulation with LPS in RAW 264.7 macrophages. Pre- and post-treatment with ALA decreased the MDA concentrations. Maximum effect was observed at 1 h pre- and post-treatment. Effects of ALA on MDA concentrations are strongly time-dependent. Results are expressed in nmol/mg of protein and represented as mean ± SEM (*n* = 3). Each experiment was performed in triplicate on three different days. A one-way ANOVA with a post hoc Tukey test was used. Statistical significance was determined at * *p* < 0.05; ** *p* < 0.01; *** *p* < 0.001; **** *p* < 0.0001; and non-significant (ns). The quantitative MDA concentration data are provided in Supplementary Table S1.

To investigate the antioxidant effects of ALA, MDA concentration was measured in LPS-stimulated macrophages (Figure 5A,B). LPS stimulation significantly increased the MDA concentration in macrophages, whereas treatment with ALA prevented this effect. Pre- and post-treatment at 1 h showed a significant decrease in MDA concentration in LPS-stimulated macrophages. Post-treatment with ALA at 3 and 6 h showed a partial protective effect on MDA concentration. These findings highlight that ALA exhibits maximum protective effects before LPS stimulation and after one hour of LPS stimulation, and these effects diminish progressively over time.

### 2.6. Effect of ALA Treatment on CSE Expression in LPS-Stimulated RAW 264.7 Macrophages

**Figure 6:**
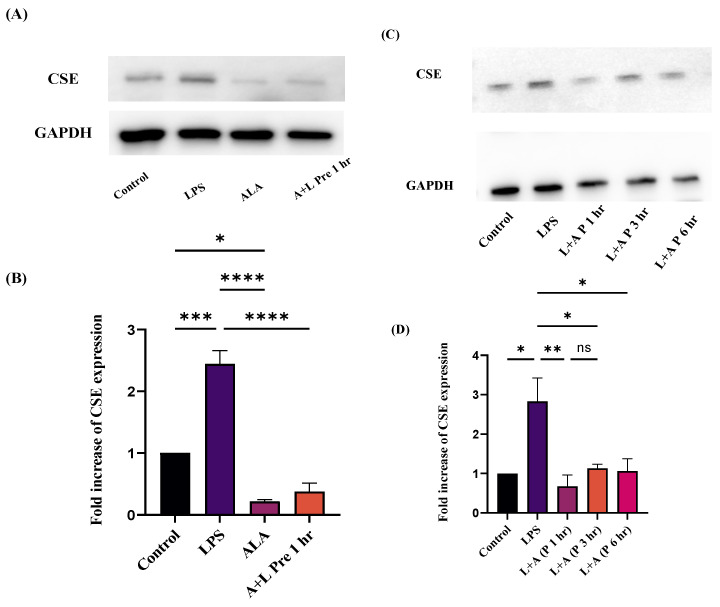
(**B**,**D**) The effects of ALA on CSE expression in LPS-stimulated RAW 264.7 macrophages. CSE expression is expressed as a fold increase over control. CSE band intensity was normalized against GAPDH. Representative Western blot images (**A**,**C**) are obtained from three independent experiments on three different days. All the data are represented as mean ± SEM (*n* = 3). A one-way ANOVA with a post hoc Tukey test was used. Statistical significance was determined at * *p* < 0.05; ** *p* < 0.01; *** *p* < 0.001; **** *p* < 0.0001; and non-significant (ns). The densitometric quantification of Western blots are provided in Supplementary Table S1.

To determine the expression of CSE in LPS-stimulated and ALA-treated cells, Western blotting was performed. CSE expression was significantly higher in LPS-stimulated cells than in the control group (Figure 6A,C). Pre-treatment with ALA significantly suppressed the CSE expression in LPS-stimulated macrophages. Post-treatment with ALA also suppressed CSE expression in LPS-treated macrophages. Maximum effect was observed after one hour, whereas a small increase in CSE expression was observed at 3 and 6 h. Glyceraldehyde-3-phosphate dehydrogenase (GAPDH) was used as a loading control to compare CSE expression.

## 3. Discussion

LPS treatment significantly increases MCP-1, IL-6, TNF-α, and IL-1β levels in macrophages and contributes to pro-inflammatory responses [14]. Results in the present study show that LPS stimulation (100 ng/mL) significantly increased the levels of MCP-1, IL-6, and TNF-α in RAW 264.7 macrophages. ALA treatment either 1 h before or 1, 3, and 6 h post-stimulation to LPS in RAW 264.7 macrophages showed an anti-inflammatory effect as evidenced by the decreased levels of MCP-1, TNF-α, and IL-6. The anti-inflammatory effects of ALA in this study are consistent with previous studies, highlighting that ALA pretreatment shows anti-inflammatory effects by reducing the levels of cytokines and chemokines after an inflammatory stimulus [15,16]. ALA showed maximum protection against LPS-induced inflammation at 1 h before LPS stimulation. However, it is important to note the protection was observed when cells were treated with ALA 1, 3, and 6 h post LPS stimulation.

Our results extend previous work by demonstrating time-dependent effects of ALA when administered after LPS stimulation. Post-treatment showed significant protection at 1 h, although these effects diminished progressively after 3 and 6 h. To the best of our knowledge, this is the first study showing the prophylactic (1 h) and therapeutic effects (1 h, 3 h, 6 h) of ALA across different time points in LPS-stimulated RAW 264.7 macrophages. This data highlights the importance of temporal dynamics of ALA-mediated amelioration of oxidative stress, inflammation, and H_2_S signaling. Administration of ALA at different time points after an inflammatory stimulus is clinically important to establish a therapeutic window for intervention. Protective effects of ALA after 1, 3, and 6 h of LPS stimulation point to the potential of ALA as a therapeutic approach after inflammation is established.

Our results indicate that LPS stimulation resulted in morphological changes in RAW 264.7 macrophages. In the present study, we found that ALA treatment preserved the morphological alterations in LPS-stimulated cells. The maximum protective effect was observed at pre-treatment. In cells in which LPS stimulation was followed by ALA treatment, significant protection in terms of morphology was observed after 1 h, this effect was progressively abolished at 3 and 6 h. These findings indicate that the effect of ALA in response to LPS exposure is time-dependent. Consistent with our results, it has been reported that normal RAW 264.7 cells are round in shape with smooth edges, whereas LPS stimulation can result in morphological changes such as elongated pseudopodia, rough edges, an increase in size, and irregular shape [17,18]. The activation of RAW 264.7 cells is characterized by the overproduction of pro-inflammatory mediators, which trigger the activation of inflammatory signaling cascades [19]. ALA administration in cecal ligation and puncture (CLP—standard method to induce sepsis in rodents) induced septic rats has been reported to significantly improve the histological architecture of the lungs and kidneys [20,21]. The accumulation of by-products of lipid peroxidation is widely studied in many pathological conditions [22]. Oxidative stress biomarkers such as F2-isoprostanes, 8-hydroxy-2′-deoxyguanosine and MDA can be used to assess the damage to nuclear components, membrane lipids, and proteins [23]. Among these biomarkers, MDA is commonly measured in biological samples to assess oxidative damage [24,25]. Several endogenous antioxidant enzymes such as superoxide dismutase (SOD), glutathione peroxidases (GPx), and CAT are also considered as crucial biomarkers in oxidative stress. ALA is an important antioxidant and provides significant protection against cell damage. Our current study showed that LPS stimulation decreased CAT activity, whereas ALA administration significantly attenuated this decrease in CAT activity. Maximum effect was observed when ALA was administered 1 h before LPS stimulation. ALA treatment at 1 h post LPS stimulation also improved CAT activity, but this effect was progressively diminished at 3 and 6 h. A similar effect was seen with MDA levels. MDA concentration in LPS-stimulated cells was significantly increased, whereas pre-treatment with ALA decreased MDA concentration. ALA post-treatment at 1 h following LPS stimulation showed decreased MDA concentration, whereas this effect was decreased progressively after 3 and 6 h. It has been reported that, in the LPS-induced septic rat model, ALA administration significantly reduced lipid peroxidation and increased free sulfhydryl group, total protein content, and SOD activities in kidney homogenates [26]. In CLP-induced septic mice, ALA treatment significantly reduced myeloperoxidase activity and lipid peroxidation in the liver. Furthermore, a significant increase in CAT activity in the liver and kidney was also observed [27].

RAW 264.7 macrophages express CSE, which serves as the main source of H_2_S production [28]. In this study, we found that LPS stimulation increased CSE expression. Our results show that both pre- and post-administration of ALA downregulate the CSE expression in LPS-stimulated macrophages. The reduced H_2_S synthesis resulting from reduced CSE expression contributes to the reduction in the production of the pro-inflammatory mediators. Our results suggest that protective effects of ALA are associated with the modulation of the CSE/H_2_S pathway. Effects of ALA on inflammatory, oxidative stress biomarkers, and CSE expression suggest that ALA may influence both upstream signaling and downstream effector pathways. Furthermore the protective effect of ALA observed after LPS stimulation, points to its potential as a therapeutic approach in already established inflammation. Several studies, from our laboratory and others, have shown that H_2_S acts as an important modulator in inflammatory diseases. H_2_S has been reported to act as a pro- and an anti-inflammatory mediator depending upon several factors such as timing of administration, dosage, and concentration. It has been reported that H_2_S can act as a pro-inflammatory mediator exacerbating inflammation, whereas slow/controlled release of H_2_S can show anti-inflammatory effects. The anti-inflammatory action of slow H_2_S releasing donors is associated with an inhibition of endogenous H_2_S synthesis possibly by a negative feedback mechanism [29,30]. A substantial body of evidence suggests that CSE inhibition can provide protective effects in inflammatory conditions. It has been shown that CSE inhibition by propargylglycine (PAG-CSE inhibitor) significantly improved the severity of acute pancreatitis and lung injury in cerulein-induced pancreatitis in mice [31]. CSE inhibition significantly reduced the levels of cytokines and chemokines in mice with sepsis and acute pancreatitis [32,33]. Furthermore, it has been reported that LPS stimulation significantly increased the activation of extra cellular signal-regulated kinase 1/2 (ERK1/2) and transcription factor nuclear factor-*κ*B (NF-*κ*B) pathway in RAW 264.7 macrophages. Whereas CSE gene silencing by small interfering RNA (siRNA) significantly suppressed the activation of these inflammatory pathways [34]. It has been found that LPS stimulation resulted in the up-regulation of CSE expression through TLR4–p38 and NF-κB pathways in both in vitro and in vivo [35]. Other studies have also shown that H_2_S acts as a pro-inflammatory mediator in LPS-induced endotoxemia in mice and rats [36,37]. In summary, these studies highlight that H_2_S could act as a pro-inflammatory mediator and exacerbate the inflammation. It is well documented that ALA confers protective effects through modulation of several interconnected signaling pathways including MAPK signaling (ERK, p38, and JNK), nuclear factor erythroid 2-related factor 2 (Nrf2)/antioxidant response element (ARE), and NF-*κ*B pathways. Studies have highlighted that ALA can exert a protective effect via H_2_S-mediated pathways. It has been reported that treatment with ALA significantly increased H_2_S formation, sulfane sulfur levels, and upregulated CSE expression in the type 2 diabetes mellitus rat model [38]. In another in vivo study, ALA treatment showed a significant reduction in paw edema in carrageenan-induced paw edema in mice. Whereas treatment with ALA and glibenclamide (an agent that blocks K_ATP_ channels, which play a crucial role in H_2_S signaling) abolished the protective effects of ALA and a significant exacerbation in paw edema was observed. This finding also indicates that the protective effects of ALA are H_2_S-mediated [39]. Our findings indicate that ALA could modulate the CSE/H_2_S pathway, providing protective effects in both prophylactic and therapeutic settings. This study showed the association of effects of ALA with the modulation of CSE expression. However, this study has a few limitations including the lack of use of H_2_S synthesis inhibitors such as PAG, siRNA gene silencing, and CSE^-/-^ gene deletion, which can provide a direct association between ALA- and CSE/H_2_S-dependent mechanisms. Furthermore, the concentration of ALA (1000 µM) was optimal for this in vitro model only; future studies are needed to demonstrate the optimal concentration for in vivo models and clinical translation.

**Figure 7:**
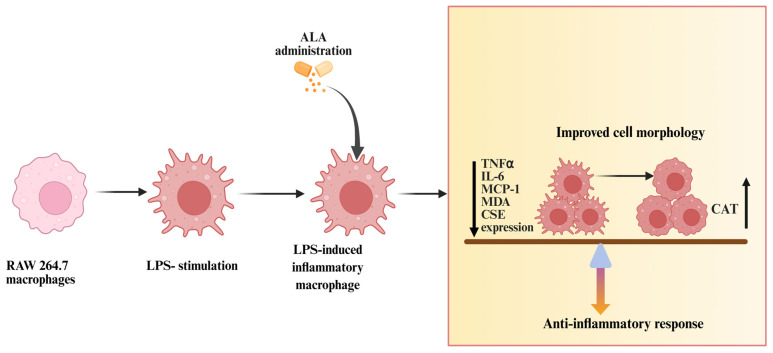
LPS stimulation significantly disrupted the morphology of RAW 264.7 macrophages. Treatment with ALA decreased the levels of TNF-α, IL-6, MCP-1, MDA, and CSE expression, increased the CAT activity, and preserved the morphology of RAW 264.7 macrophages.

In conclusion (Figure 7), in the present study, we have demonstrated for the first time that ALA significantly protects against cell injury in LPS-stimulated macrophages at both pre- and post-treatment at different time points. Although the role of ALA in several diseases has been reported, the underlying mechanisms remain unclear. The striking findings of this study are that ALA preserved cell morphology, decreased the levels of cytokines, chemokines, and MDA and increased the CAT activity by modulating the CSE/H_2_S pathway in a time-dependent manner. These results are a significant contribution to our understanding of the mechanisms by which ALA contributes to the mitigation of inflammation and could contribute to the management of different inflammatory conditions such as sepsis.

## 4. Materials and Methods

### 4.1. Cell Culture

RAW 264.7 cells derived from murine macrophages—obtained from American Type Culture Collection (ATCC) were maintained at 37 °C at 5% CO_2_ in Dulbecco’s modified Eagle’s medium (DMEM) (ThermoFisher Scientific, Waltham, MA, USA) supplemented with 10% fetal-bovine serum (heat-inactivated) and 100 U/mL penicillin and streptomycin (Gibco; ThermoFisher Scientific, Waltham, MA, USA). Cells were passaged every week, and media changed twice a week after washing with sterile PBS. Prior to the study, RAW 264.7 cells were enzymatically detached using 0.25% trypsin (Gibco; ThermoFisher Scientific, Waltham, MA, USA) and seeded in a 12-well plate at 2 × 10^5^ cells per well.

### 4.2. Treatment with LPS and ALA

Based on preliminary findings, the LPS concentration of 100 ng/mL was considered the optimum concentration for subsequent experiments. LPS was purchased from Sigma-Aldrich (St. Louis, MO, USA) and dissolved in sterile PBS for the treatment of RAW 264.7 macrophages. ALA (Cayman Chemical, Ann Arbor, MI, USA) was dissolved in sterile dimethyl sulfoxide (DMSO, Sigma-Aldrich, St. Louis, MO, USA) to prepare stock solutions. The stock solution was further diluted in PBS for cell treatment. To prevent cytotoxicity, the final concentration of DMSO was maintained at less than 0.05% in the culture medium. To assess the anti-inflammatory effects of ALA, different concentrations of ALA (300 µM, 500 µM, 1000 µM, and 1500 µM) were used. The maximum effect was observed at 1000 µM, and this concentration was used for subsequent experiments. Cells were categorized into the following experimental groups: control group, inflammation (LPS) model, pre-treatment with ALA (1 h), and post-treatment with ALA after LPS stimulation at different time points (1 h, 3 h, and 6 h). Control cells were cultured with DMSO and PBS only. After treatment, the cells were incubated for 24 h, and then the cell supernatants and pellets were carefully harvested for further experiments.

### 4.3. Enzyme-Linked Immunosorbent Assay (ELISA) Analysis

To investigate the levels of several pro-inflammatory mediators, including TNF-α, IL-6, and MCP-1, a sandwich ELISA was performed in cell supernatant. The levels of these pro-inflammatory mediators were measured by using specific DuoSet ELISA Kits (R&D System Minneapolis, Minneapolis, MN, USA) according to the manufacturer’s instructions. The concentrations of these mediators were corrected for the total protein content by Bradford protein assay.

### 4.4. Morphology of RAW 264.7 Macrophages

Raw 264.7 macrophages were cultured and treated at different time points as described above. Morphological changes were observed using a light microscope (inverted Leica light microscope). Representative images were taken at 10× magnification. A simple ImageJ-based morphometric analysis was performed to support the qualitative analysis of cells. Approximately twenty cells from each field were selected manually using ImageJ. The mean area of cells from each field was calculated from measurements (obtained from one experiment). The data are presented as raw values in a Supplementary Excel File S1.

### 4.5. Measurement of MDA and Catalase CAT Concentrations in Cell Lysates

The concentrations of MDA (BQC Redox Technologies, Asturias, Spain) and CAT (ThermoFisher Scientific, Waltham, MA, USA) were measured in the cell lysates according to the manufacturer’s instructions. The concentrations of these mediators in cell lysates were corrected for the total protein content by Bradford protein assay.

### 4.6. Western Blot Analysis

RAW264.7 macrophages were cultured in DMEM media and stimulated using LPS with or without ALA (1 h pre-treatment with ALA and post-treatment at 1, 3, and 6 h). Western blotting was performed by following the same protocol with minor modifications [40]. CSE protein concentration was quantified by using Direct Detect^®^ assay-free infrared quantification system using single-use Direct Detect cards (MilliporeSigma, Burlington, MA, USA). The cells were lysed by using a cold radioimmunoprecipitation assay (RIPA) buffer-supplemented with protease inhibitors. Equal amounts of protein (25 µg) were separated by electrophoresis using a 12% sodium dodecyl sulfate-polyacrylamide gel. Then they were transferred onto a polyvinylidene difluoride (PVDF, Bio-Rad, Hercules, CA, USA) membrane via wet transfer for 50 min. The membrane was blocked with blocking buffer (containing 5% non-fat dry milk) for 1 h at room temperature. After blocking, the membrane was incubated overnight at 4 °C with the primary antibodies CTH (1:2000; mouse monoclonal antibody; Abnova cat. no. H00001491-M01) and GAPDH (1:10,000; rabbit polyclonal antibody; ThermoFisher catalog no PA1-987). After overnight incubation, the membrane was washed and incubated with corresponding secondary antibodies (Goat-anti-mouse HRP, and Goat-anti-rabbit HRP; Santa Cruz) for one hour at room temperature and detected with chemiluminescent reagent (Supersignal West Pico, Thermo Scientific, Waltham, MA, USA). Bands were visualized by using a Chemi-doc instrument (Uvitec, Cambridge, UK).

### 4.7. Statistical Analysis

All the data presented in this study are reported as mean ± standard error of measure (SEM). To compare the differences between various groups, a one-way ANOVA followed by post hoc Tukey’s test was performed. GraphPad Prism software (version 10.6.1 (892), San Diego, CA, USA) was used to perform statistical analysis in this study. Statistical significance was defined as * *p* < 0.05; ** *p* < 0.01; *** *p* < 0.001; **** *p* < 0.0001; and non-significant (ns).

## Data Availability

All data generated or analyzed during this study are included in this published article.

## Supplementary Materials

The following supporting information can be downloaded at: https://www.mdpi.com/article/10.3390/ijms27020949/s1.
