# Supplementary material for: Anti-Inflammatory Effects of Alpha-Lipoic Acid Modulate Cystathionine-γ-Lyase Expression in RAW 264.7 Macrophages
Source: Int J Mol Sci. 2026 Jan 18;27(2):949. doi: 10.3390/ijms27020949 (PMC12842497; doi:10.3390/ijms27020949)
Supplement: Supplementary file 1 [file ijms-27-00949-s001.zip › Supplementary Table.pdf]

Table S1: Effects of ALA on different inflammatory and oxidative stress biomarkers at different time-points. Data is presented as mean ± SEM from three independent experiments. LPS-stimulation significantly increased the levels of biomarkers relative to control group. This increase was modulated by ALA administration across different time-points.

**Biomarker**	**Control**	**LPS**	**A+L Pre 1 hr**	**A+L Post 1 hr**	**A+L Post 3 hr**	**A+L Post 6 hr**
TNF-α pg/mg	Pre: 1076 ± 246.5<br>Post: 1538 ± 155.3	Pre: 30849 ± 5135<br>Post: 34456 ± 4305	4333 ± 1013	13749 ± 3337	13435 ± 2356	16941 ± 819.1
IL-6 pg/mg	Pre: 393.2 ± 107.9<br>Post: 726 ± 270	Pre: 9087 ± 897<br>Post: 9405 ± 1028	1580 ± 224.2	3203 ± 413.6	3201 ± 545.1	4477 ± 407.6
MCP-1 pg/mg	Pre: 83.70 ± 4.20<br>Post: 95.93 ± 11.90	Pre: 61392 ± 2953<br>Post: 59049 ± 3185	2054 ± 286.8	8297 ± 2718	14023 ± 2951	29627 ± 4034
MDA nmol/mg	1.53 ± 0.40	4.38 ± 0.45	1.81 ± 0.48	1.39 ± 0.24	1.91 ± 0.15	2.80 ± 0.22
CAT mU/mg	5.29 ± 0.42	2.52 ± 0.23	12.08 ± 1.68	6.25 ± 0.17	4.46 ± 0.24	3.36 ± 0.23
CSE expression	Pre: 1<br>Post: 1	Pre: 2.44 ± 0.21<br>Post: 2.82 ± 0.59	0.37 ± 0.13	0.67 ± 0.28	1.13 ± 0.10	1.16 ± 0.31
